# Supplementary material for: A major locus confers triclabendazole resistance in Fasciola hepatica and shows dominant inheritance
Source: PLoS Pathog. 2023 Jan 26;19(1):e1011081. doi: 10.1371/journal.ppat.1011081 (PMC9904461; doi:10.1371/journal.ppat.1011081)
Supplement: S8 Table — (DOCX) [file ppat.1011081.s008.docx]

**Table S8. Differential gene expression of the candidate genes throughout the *Fasciola hepatica* life cycle, based on average TPM values.**

| Gene no.^1^ | Gene id (scaffold id in bold) | Predicted Protein Description^2^ | Met^3^ | NEJ1hr | NEJ3hr | NEJ24hr | Immature | Adult | Egg |
| --- | --- | --- | --- | --- | --- | --- | --- | --- | --- |
| 1  2 | maker-scaffold10x_**1853**_pilon-snap-gene-0.14  (maker-scaffold10x_**1853**_pilon-snap-gene-0.15) | 26S proteasome non-ATPase regulatory subunit 14 | 250.19  8.50 | 249.92  5.26 | 266.90  4.54 | 278.81  14.82 | 283.01  55.65 | 274.48  14.92 | 192.25  17.14 |
| 3 | maker-scaffold10x_**1853**_pilon-snap-gene-0.13 | Uncharacterised protein | 19.18 | 20.30 | 80.78 | 30.29 | 52.91 | 510.87 | 337.53 |
| 4 | maker-scaffold10x_**157**_pilon-snap-gene-0.196 | EGF-like protein | 5.10 | 4.96 | 4.19 | 4.86 | 18.75 | 0.73 | 1.64 |
| 5 | maker-scaffold10x_**157**_pilon-snap-gene-0.179 | Putative multidrug resistance protein 1, 2, 3 (P glycoprotein 1, 2, 3); ATP binding cassette subfamily B MDR TAP | 0.04 | 0.06 | 0.10 | 0.02 | 4.86 | 0.32 | 0.31 |
| 6 | maker-scaffold10x_**157**_pilon-snap-gene-0.180 | SANT/Myb-like DNA-binding domain protein | 28.86 | 24.03 | 30.15 | 47.58 | 68.80 | 24.72 | 11.27 |
| 7 | maker-scaffold10x_**157**_pilon-snap-gene-0.197 | ADP-ribosylation factor 2 | 315.38 | 330.69 | 363.17 | 308.15 | 235.88 | 475.38 | 153.57 |
| 8 | maker-scaffold10x_**157**_pilon-snap-gene-0.181 | RNA-binding protein sym-2/ Heterogeneous nuclear ribonucleoprotein | 8.97 | 6.27 | 6.01 | 11.05 | 23.40 | 5.75 | 2.29 |
| 9 | maker-scaffold10x_**157**_pilon-snap-gene-0.198 | DNA directed RNA Polymerase I and III (A/C) shared subunit | 14.21 | 14.88 | 14.15 | 22.23 | 14.18 | 23.56 | 12.17 |
| 10 | maker-scaffold10x_**157**_pilon-snap-gene-0.182 | Ras-related protein Rap-1 | 141.86 | 167.55 | 168.63 | 159.78 | 222.68 | 168.94 | 37.83 |
| 11 | maker-scaffold10x_**157**_pilon-snap-gene-0.183 | Receptor protein serine/threonine kinase | 12.24 | 13.52 | 13.18 | 29.29 | 47.05 | 23.51 | 0.45 |
| 12 | maker-scaffold10x_**157**_pilon-augustus-gene-0.97 | D-amino-acid oxidase/ D-aspartate oxidase | 17.57 | 17.89 | 15.47 | 14.94 | 64.93 | 13.22 | 118.24 |
| 13 | maker-scaffold10x_**157**_pilon-snap-gene-0.184 | Max-like protein X | 302.14 | 317.44 | 278.76 | 162.66 | 188.36 | 286.38 | 113.86 |
| 14 | maker-scaffold10x_**157**_pilon-snap-gene-0.185 | EGF-like protein | 0.56 | 0.59 | 1.75 | 0.10 | 17.33 | 2.77 | 0.04 |
| 15 | maker-scaffold10x_**157**_pilon-snap-gene-0.186 | Surfeit locus protein 4 | 48.62 | 54.85 | 54.56 | 68.19 | 12.20 | 54.43 | 23.83 |
| 16 | augustus_masked-scaffold10x_**157**_pilon-processed-gene-0.14 | TFIIH basal transcription factor complex helicase XPD subunit | 7.54 | 4.96 | 4.51 | 5.24 | 14.24 | 4.26 | 12.05 |
| 17 | maker-scaffold10x_**157**_pilon-snap-gene-0.187 | Fatty acid binding protein V | 11.83 | 9.56 | 10.01 | 30.72 | 170.27 | 54.03 | 58.37 |
| 18 | maker-scaffold10x_**157**_pilon-snap-gene-0.200 | **Stomatin-2 / SPFH Domain / Band 7 family protein** | 141.16 | 134.19 | 166.95 | 204.82 | 60.08 | 16.06 | 3.44 |
| 19 | maker-scaffold10x_**157**_pilon-snap-gene-0.201 | Glycosylphosphatidylinositol (GPI) ethanolamine phosphate transferase 1 | 42.77 | 40.04 | 36.64 | 49.06 | 80.71 | 40.48 | 6.45 |
| 20 | maker-scaffold10x_**157**_pilon-pred_gff_StringTie-gene-0.138 | Sugar phosphate exchanger 3 | 6.36 | 4.60 | 6.32 | 11.50 | 13.37 | 17.81 | 30.54 |
| 21 | maker-scaffold10x_**157**_pilon-snap-gene-0.203 | Ribonuclease 3 | 3.66 | 3.54 | 7.66 | 11.05 | 24.45 | 10.18 | 13.16 |
| 22 | maker-scaffold10x_**157**_pilon-snap-gene-0.188 | Putative serine-rich repeat protein | 0.74 | 0.46 | 0.72 | 1.88 | 97.81 | 20.75 | 0.31 |
| 23 | maker-scaffold10x_**157**_pilon-snap-gene-0.204 | Putative transferase CAF17, mitochondrial | 13.15 | 12.08 | 15.20 | 19.59 | 14.43 | 12.80 | 4.64 |
| 24 | maker-scaffold10x_**157**_pilon-snap-gene-0.205 | Lamin-1/ Neurofilament protein | 8.38 | 8.56 | 8.09 | 7.23 | 31.94 | 2.72 | 29.65 |
| 25 | maker-scaffold10x_**157**_pilon-snap-gene-0.189 | Gyf domain protein | 54.30 | 48.41 | 45.84 | 34.65 | 47.23 | 83.19 | 79.08 |
| 26 | snap_masked-scaffold10x_**157**_pilon-processed-gene-0.72 | Prominin | 5.82 | 6.59 | 3.79 | 2.25 | 9.21 | 7.94 | 0.47 |
| 27 | maker-scaffold10x_**157**_pilon-snap-gene-0.206 | Phospholipid transport protein / CRAL-TRIO / SEC14-like | 64.17 | 70.08 | 54.30 | 50.76 | 32.97 | 105.17 | 229.51 |
| 28 | maker-scaffold10x_**157**_pilon-snap-gene-0.190 | Ubiquitin carboxyl-terminal hydrolase | 27.45 | 32.13 | 19.42 | 10.44 | 9.75 | 92.95 | 32.95 |
| 29 | maker-scaffold10x_**157**_pilon-snap-gene-0.207 | Ubiquitin carboxyl-terminal hydrolase | 28.08 | 29.91 | 19.49 | 11.27 | 12.74 | 70.59 | 22.92 |
| 30 | maker-scaffold10x_**157**_pilon-augustus-gene-0.89 | Ubiquitin carboxyl-terminal hydrolase | 191.84 | 182.65 | 194.29 | 136.39 | 70.75 | 126.03 | 43.30 |

^1.^ Gene number corresponds with Fig. 5. ^2.^ Protein description and function were determined using UniProt Blast, WormBase ParaSite Version 14 Blast, OrthoDB version 9, and InterPro ^3.^ Life cycle stages: Met, metacercariae; NEJ 1hr, newly excysted juvenile (NEJ) 1 hr post-excystment; NEJ 3hr, NEJ 3hr post-excystment; NEJ 24hr, NEJ 24hr post-excystment; Immature, immature fluke 21 days post-infection.
