# Supplementary material for: A major locus confers triclabendazole resistance in Fasciola hepatica and shows dominant inheritance
Source: PLoS Pathog. 2023 Jan 26;19(1):e1011081. doi: 10.1371/journal.ppat.1011081 (PMC9904461; doi:10.1371/journal.ppat.1011081)
Supplement: Attachment — Submitted filename: Response to Reviewers Comments.docx [file ppat.1011081.s013.docx]

15^th^ December 2022

**Re. A major locus confers triclabendazole resistance in Fasciola hepatica and shows dominant inheritance**

Dear Guest Editor, Section Editor and Editors-in-chief,

We would like to thank all independent reviewers and the editorial board for their careful review of our manuscript and their positive comments about its content and quality. As requested, we have provided additional discussion of how future work will pinpoint the gene involved in triclabendazole resistance in *Fasciola hepatica* and hope that it will now be accepted for publication in PLOS Pathogens.

Whilst it was made clear that additional experiments were not essential to its acceptance, we had already been working on some of the aspects raised by Reviewer 3 and we have included an additional two tables of supplemental data for completeness.

We have made all other corrections raised by the four reviewers and we have provided a revised manuscript highlighting the edited text, a list of all the changes in response to reviewers and a clean version of the revised manuscript as requested.

We hope our manuscript meets with your satisfaction,

Yours sincerely,

Nicola Beesley

**Response to Reviewers**

**Issues raised under Part I – Summary and Part II – Major Issues: Key Experiments Required for Acceptance**

Issues were raised by Reviewer #3 and have been addressed as follows:

*Reviewer 3: ‘However, further discussion of how future work will narrow down the gene involved and the validation approaches (e.g. RNAi) that can be used should be included in this paper.*

*I’d suggest some further exploration of these candidate genes. For example, genes that are not expressed in adult worms are unlikely to be involved and could be excluded. Genes that are invariant, or have only synonymous mutations can be deprioritized (with the caveat that non-coding changes may also be involved), while genes carrying non-synonymous changes with the expected segregation pattern can by prioritized. I suggest including a table or supplement showing the amino acid changes segregating in crosses within candidate loci, and in related flukes for the genes 3-10 in table 4. This would be informative for readers, and helpful for determining possible causative genes. For example, if a candidate gene is conserved across related flukes, but shows non-synonymous change in segregating resistant alleles, this would be of particular interest.’*

**Response: We have been exploring gene expression and sequence variation in our 30 candidate genes and we are happy to provide further analysis showing these data. We have provided two additional tables in the supplementary materials. The first additional table (S8 Table) details expression data for the 30 candidate genes using publicly available RNA-seq datasets. The second table (S9 Table) focuses on amino acid changes segregating in crosses within candidate loci for genes 3-10. The text in the results (lines 267-288), discussion (lines 342-344, 358-362, 366-374, and 400-403) and materials and methods (lines 621-628) has been revised to reflect inclusion of these data.**

*Reviewer 3 ‘Copy number variants often underlie overexpression of ABC transporters – does the genomic data provide any evidence for this? Similarly, overexpression of genes within the QTL could be used to identify prime candidates. As the number of genes is quite small (30), systematic gene knockdown experiments with RNAi could be used – this gene manipulation approach is possible for Fasciola (*[*https://www.ncbi.nlm.nih.gov/pmc/articles/PMC5749881/*](https://www.ncbi.nlm.nih.gov/pmc/articles/PMC5749881/)*) and should be mentioned. The paper would be strengthened by inclusion of a further discussion section detailing what strategies can be used to validate the causative resistance loci and variants … Some discussion of what dominant inheritance suggests about the mechanism of resistance could also be useful.’*

**Response: We have added a sentence to confirm that no copy number variants of the ABC transporter are evident in our genomic data, based on our current annotation (see lines 280-282). Comprehensive expression studies are underway in our laboratory to identify if any of the 30 genes are under/over expressed in *F. hepatica* adult and juvenile parasites, +/- triclabendazole exposure, but are not sufficiently advanced for us to comment at this stage. We have included additional sections in the discussion on the use of RNAi (lines 404-417), and we have raised the issue of what dominant inheritance suggests about the mechanism of inheritance (lines 349-352).**

*‘Reviewer 3: Methodology clarification needed: Ln 202-204 states: “We genotyped 249 x F2 TCBZ+ and 485 x F2 TCBZ- parasites (S3 Table) with a subset of 48 SNPs derived from each of the six mapped scaffolds and 16 SNPs from neutral (not under selection) scaffolds of comparable size” It would be best to do this analysis with only 485 x F2 TCBZ- (untreated) parasites, as this will allow them to investigate segregation without drug selection.’*

**Response: We thank Reviewer 3 for highlighting this, genotyping of these parasites was used for two purposes (1) to determine whether these scaffolds were linked and (2) to determine inheritance patterns. We have now made it clearer in the text which parasites were used for which purpose (lines 200, 224-225, and 573-577). In response to this suggestion, we have repeated the linkage analysis to include only parasites from untreated hosts (lines 204-206) and we include an updated Figure 3 (lines 1168-1175). The interpretation and conclusions that can be drawn based on this revised figure remain unchanged from the original, but the figure is more precise based on removal of drug-selected parasites from the analysis.**

**Issues raised under Part III – Minor Issues: Editorial and Data Presentation Modifications**

**Reviewer #1: Some minor comments for attention of authors:**

*1. ‘The segment in the discussion that summarises the main data implicating pgps in flukicide resistance needs some attention. For example, under (b) the context for the role of ivermectin (potentially as a competitive substrate for pgp activity) will not be clear for the reader and so needs some explanation. Further, the third piece of evidence under (c) is incomplete, appearing to be accidentally truncated at pgp – the authors may have meant the pgp inhibitor verapamil(R-)?*

**Response: Corrected (lines 348-362).**

*2. On lines 388 and 392, where genes previously implicated in TCBZ resistance are being ruled out because of their absence from the resistance loci, the authors should consider defining that these genes are being ruled out as the basis for TCBZ resistance in the fluke clonal and field populations studied within the MS.’*

**Response: Corrected (lines 440 and 451).**

*3. L414 ‘ as prime candidates for conferring’….*

**Response: Corrected (line 473).**

*4. ‘To be considered by authors, but not essential to publication:

Amongst the 30 genes identified, the potential role of the ABCB1 gene in TCBZ resistance is intriguing. Notably, a cursory glance at its expression in liver fluke transcriptomic datasets suggests very low (or no) expression in juvenile liver fluke. This could diminish the likelihood of that specific gene being a key player, assuming that the resistance phenotype in question is not confined to the adult worm. The clonal resistant isolates used in the study were exposed to TCBZ treatments during patent infections, but it is unclear if there are data on the susceptibility of the juveniles? If the authors know that the resistant trait is present in both the adult and juvenile worms (or indeed is restricted to the adult), this could provide impetus for looking at stage-specific expression patterns of the 30 ‘candidate’ genes to see if any are expressed in line with the resistance phenotype.

Did the authors consider if the locus highlighted in the study contained any non-coding genes and/or any known binding sites for known miRNAs? If not this could be highlighted as something for consideration in future work.’*

**Response: We thank Reviewer 1 for their comments. As stated above in response to Reviewer 3, comprehensive expression studies are underway in our laboratory to identify if any of the 30 genes are under/over expressed in *F. hepatica* adult and juvenile parasites, +/- triclabendazole exposure, but are not sufficiently advanced for us to comment at this stage. However, we have included expression data for the 30 candidate genes using publicly available RNA-seq datasets (S8 Table; lines 267-279) and added to the discussion to reflect the need to address stage specific mechanisms of resistance (see lines 404-412). Similarly, we have highlighted that interrogating the locus for known binding sites for miRNAs is an interesting avenue for future work (see lines 412-416).**

**Reviewer #3**

*5.* *Ln 58. “Our study is the first of its kind for liver fluke.” I suggest removing this statement – this is not needed to sell the science. Likewise, I’d remove “We have delivered several firsts” (Conclusion – ln 405).*

**Response: Removed (line 58 and line 464).**

*6. Ln 83-83: “As a result, enhanced molecular surveillance of resistance allele frequencies in field populations has facilitated targeted treatments with efficacious drugs and reduced selection pressure for resistance development [22]”
This is somewhat overstated – the discovery of SmSULT-OR has allowed molecular surveillance for OXA-R alleles in natural populations, but this is of limited utility from a practical standpoint, because OXA is no longer used to treat schistosome infections. I’d change to “This has allowed global mapping of OXA-R alleles in natural populations” and you could also cite* [*https://pubmed.ncbi.nlm.nih.gov/31652296/*](https://pubmed.ncbi.nlm.nih.gov/31652296/)

**Response: We thank the reviewer for this correction and have revised the sentence as suggested (lines 82-83)**

*7. It is strange that several prior candidate genes all fall in the QTL region. My expectation is that just one of these genes is driving the QTL. This would be worth addressing in the discussion. See ln 256-261 “…namely an ADP ribosylation factor (Gene 7: ARF, maker256 scaffold10x_157_pilon-snap-gene-0.197; Fig. 5; Table 4), a Ras-related protein (Gene 10: 257 Ras-RP, maker-scaffold10x_157_pilon-snap-gene-0.182; Fig. 5; Table 4), an ABCB1 gene 258 (Gene 5: ABCB1, maker-scaffold10x_157_pilon-snap-gene-0.179; Fig. 5; Table 4). Lying 259 within the mapped locus, albeit slightly outside the strongest signal of selection, is another 260 gene that has been the focus of candidate gene studies for TCBZ resistance, a FABP V gene 261 (Gene 17: FABPV, maker-scaffold10x_157_pilon-snap-gene-0.187; Fig. 5; Table 4).”*

**Response: We agree with this reviewer, we also think that just one gene is driving the QTL. Throughout this work our approach has been to take an unbiased perspective in what drives triclabendazole resistance and we merely wished to highlight that one of several candidates may be involved. We also did not wish to speculate further which gene is responsible, or the most likely candidate. However, in response to Reviewers 1 and 3, we have happily provided additional observations to allow prioritisation of candidates (lines 267-288; S8 Table and S9 Table) and we have given a discussion of future work to pinpoint the gene (lines 404-417). This should clarify the issue raised here.**

*8. Ln 613: “and stimulated to shed cercariae onto visking tubing” . Please explain or add reference for the non-Fasciola audience.*

**Response: We have expanded this sentence and included a reference to our previous studies that give further detail (lines 680-682).**

**Reviewer #4**

*9. A grammatically better title might be: “A single major locus confers a dominantly inherited trait conferring triclabendazole resistance in Fasciola hepatica”*

**Response: We have amended the title for the manuscript to ‘A major locus confers triclabendazole resistance in *Fasciola hepatica* and shows dominant inheritance’ (lines 1-2)**

*10. Figure 5 : Can the authors suggest why the LRT signals across the 3.2 Mbp locus for the experimental cross and field data are adjacent rather than more coincident*

**Response: We now offer possible explanation for why this might be the case in the discussion (lines 418-427), importantly we now have the tools to explore this further.**

*11. Line 217 I think “Future” should read “Further”*

**Response: Corrected (line 215).**
